# A novel polyethylene glycol (PEG)‐drug conjugate of Venetoclax, a Bcl‐2 inhibitor, for treatment of acute myeloid leukemia (AML)

**DOI:** 10.1002/cnr2.1485

**Published:** 2021-06-26

**Authors:** Hidenori Ando, Yuta Murakami, Kiyoshi Eshima, Tatsuhiro Ishida

**Affiliations:** ^1^ Department of Pharmacokinetics and Biopharmaceutics, Institute of Biomedical Sciences Tokushima University Tokushima Japan; ^2^ Biotechnology & Medical Division, Planning Department Sanyo Chemical Industries, Ltd Kyoto Japan; ^3^ Headquarters, Delta‐Fly Pharma Inc. Tokushima Japan

**Keywords:** acute myeloid leukemia (AML), PEG‐drug conjugate, polyethylene glycol (PEG), Venetoclax

## Abstract

**Background:**

Venetoclax (VTX) is an anticancer drug. It is a selective Bcl‐2 inhibitor that is clinically used for the treatment of patients with lymphomas and leukemias. Treatment with VTX, however, is accompanied by severe adverse events such as tumor lysis syndrome and neutropenia, because VTX readily binds to serum proteins, which results in poor pharmacokinetics and poor tumor tissue concentration. To avoid such adverse events, VTX is administered using a daily or weekly ramp‐up schedule that is cumbersome in clinical situations.

**Aims:**

To overcome these shortcomings, we prepared a novel polyethylene glycol (PEG)‐drug conjugate of VTX (PEG‐VTX) and evaluated its cytotoxic effects on acute myeloid leukemia (AML) both in vitro and in vivo.

**Methods and results:**

VTX and 4‐armed PEG derivatives were covalently attached through an amide bond linker. In a series of in vitro studies, PEG‐VTX selectively induced potent growth inhibition of MV4‐11 human AML cells via the inducement of Bcl‐2‐mediated apoptosis. PEG‐VTX had the effect of free VTX, presumably due to the protease‐mediated release of VTX from the conjugates. In in vivo studies with AML tumor‐xenograft mice models, intravenous PEG‐VTX promoted sufficient tumor growth suppression. Compared with a regimen of oral free VTX, the intravenous regimen in those studies used a VTX dosage that was 15–30 times smaller for an OCI‐AML‐2 xenograft model and a dosing regimen that was less frequent for an MV4‐11 xenograft model. The most important development, however, was the absence of weight loss related to severe side effects throughout the treatments. An increase in water solubility and the resultant hydrodynamic size of VTX via PEGylation improved the pharmacokinetics of VTX by avoiding protein interactions and lessening the extravasation from blood. The result was an increase in tumor accumulation and a decrease in the nonspecific distribution of VTX.

**Conclusion:**

The results of this study suggest that PEG‐VTX could be an alternative therapeutic option for the safe and effective treatment of patients with AML.

## 1 INTRODUCTION

Apoptosis, or programmed cell death, is a pivotal process that removes damaged and/or unnecessary cells in the human body. In the environment of a tumor, however, defects can occur at any point along apoptotic signaling pathways and can lead to oncogenesis, tumor proliferation and metastasis, and chemoresistance.^1^ The B‐cell leukemia/lymphoma 2 (Bcl‐2) protein was the first apoptotic regulator identified as an agent that blocks the mitochondrial apoptotic pathway^2^, ^3^, ^4^ by abrogating the oligomerization of pro‐apoptotic proteins, such as Bcl‐2‐associated X protein (Bax) and Bcl‐2 antagonist killer 1 (Bak).^5^ Several clinical reports have described how the Bcl‐2 gene is overexpressed in 84% of patients with acute myeloid leukemia (AML) at the time of diagnosis and in almost all (95%) patients who experience a relapse.^6^ Overexpression of the Bcl‐2 gene is associated with poor prognosis, a low complete remission (CR) rate, a short survival time, and chemoresistance.^7^, ^8^ Accordingly, inhibition of the Bcl‐2‐triggered suppression of apoptosis is expected to be a promising therapeutic approach for patients with AML via inducing an effective programming of the death of cancer cells.

To date, several anticancer agents have been developed to target Bcl‐2‐family proteins. Obatoclax (GX15‐070) is a broad inhibitor of Bcl‐2 family proteins such as Bcl‐2, Bcl‐xL, Bcl‐w, and Mcl‐1,^9^ and is currently undergoing a phase I study for the treatment of patients with chronic lymphocytic leukemia (CLL).^10^ Although Obatoclax has produced modest clinical outcomes, it seems to cause undesired severe neurological events.^10^ Navitoclax (ABT‐263) is the first orally bioavailable inhibitor against Bcl‐2‐family proteins, including Bcl‐2, Bcl‐xL, and Bcl‐w.^11^, ^12^ In clinical trials, Navitoclax has produced good therapeutic outcomes for both patients with lymphoid malignancies^13^ and with CLL,^14^ but it caused thrombocytopenia, which is a severe undesired side effect that is frequently observed in Bcl‐xL. Therefore, Navitoclax was precluded from further clinical trials. In order to avoid such severe side effects due to Bcl‐2 nonselective inhibition, Venetoclax (VTX; ABT‐199), a selective Bcl‐2 inhibitor, has been developed.^15^ In clinical trials, VTX has produced substantial therapeutic outcomes in patients with lymphomas and was finally approved and marketed as VENCLEXTA.^16^

Nevertheless, VTX continued to cause several adverse effects, such as tumor lysis syndrome (TLS) and neutropenia.^17^ VTX is highly bound to serum proteins (>99.9%) in blood circulation,^16^ which causes poor pharmacokinetics of VTX and subsequent severer adverse events. In the clinical trials of VTX, laboratory TLS, which is defined as an increase in uric acid, potassium, phosphorus, or calcium, was observed in 5.7% of patients with lymphoma. A small percentage (2.7%) of patients with clinical and laboratory TLS tend to experience one or more of the following adverse side effects: an increase in creatinine, cardiac arrhythmia or sudden death, and/or seizure.^18^, ^19^ In clinical trials, treatment with VTX has caused grade 3/4 adverse events that include neutropenia (39.6%), thrombocytopenia (29.2%), infection (25.0%), neutropenic fever (7.9%), and diarrhea (6.9%).^19^ To overcome such serious adverse events, VTX is administered to patients with AML using a cumbersome daily ramp‐up schedule over 4 days from a starting dose (100 mg/body) to the recommended dose (400 or 600 mg/body).^16^ In addition, guidance for VTX treatment has been provided to respond to a decrease in neutrophil count via the use of granulocyte colony stimulating factor (G‐CSF), dose interruption, or dose reduction.^20^ Due to such poor pharmacokinetics and subsequent severe side effects, other approaches are still required in order to improve the usability and efficacy of VTX in clinical settings.

Polyethylene glycol (PEG) is a nonionic synthetic polymer and an excellent tool that can impart favorable pharmacokinetic and pharmacodynamic characteristics to drugs with a low molecular weight.^21^, ^22^ Several PEG‐anticancer agent conjugates such as PEGylated SN38 (EZN‐2208)^23^, ^24^, ^25^ and PEGylated irinotecan (NKTR‐102)^26^, ^27^, ^28^, ^29^ have been in clinical trials. Conjugation of anticancer agents with PEG improves the water solubility and dispersibility of the agents as well as increasing their molecular weights. Following intravenous injection, the resultant PEG‐anticancer agent conjugates show prolonged blood circulation via the avoidance of glomerular filtration due to an increase in molecular weight^22^ and by preventing interactions with serum proteins by increasing the water solubility. The long circulation properties gained by PEGylation allow the PEG‐conjugates extensive accumulation in solid tumors via enhancements in permeability and retention (EPR).^30^, ^31^ These effects led us to assume that the conjugation of VTX with PEG could improve the usability and efficacy of VTX, although the PEG‐drug conjugate of VTX must be injected intravenously.

In this study, therefore, we designed and synthesized a novel PEG‐drug conjugate of VTX (PEG‐VTX) using a 4‐armed PEG derivative. In our synthetic design, VTX was covalently attached to each of the terminal ends of a 4‐armed PEG derivative with an amide bond linker. The cytotoxicity of PEG‐VTX was studied in vitro using a human AML cell line. Tumor growth suppression and body weight changes in AML tumor‐xenograft mouse models were monitored during treatments with intravenous PEG‐VTX and oral free VTX.

## 2 MATERIALS AND METHODS

### 2.1 Materials

VTX (4‐[4‐[[2‐(4‐chlorophenyl)‐4,4‐dimethylcyclohexen‐1‐yl]methyl]piperazin‐1‐yl]‐*N*‐[3‐nitro‐4‐(oxan‐4‐ylmethylamino)phenyl]sulfonyl‐2‐(1*H*‐pyrrolo[2,3‐b]pyridin‐5‐yloxy)benzamide) (M.W. 868.44) was purchased from Acesys Pharmatech. PEG derivative (4‐armed PEG, M.W. 40 000 Da) was purchased from Sanyo Chemical Industries. All other reagents were of analytical grade.

### 2.2 Chemical synthesis and analysis of PEG‐VTX

**FIGURE 1:**
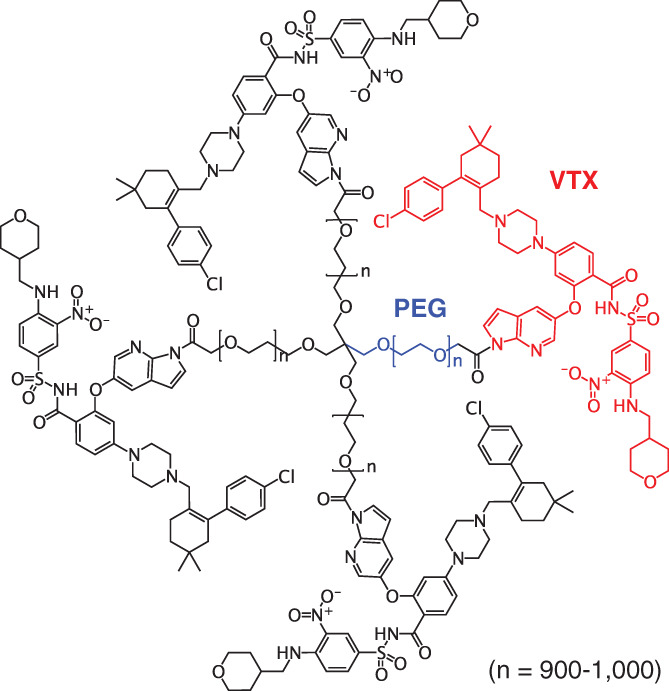
Chemical structure of synthesized PEG‐VTX

Under a N_2_ atmosphere, PEG derivative (4.530 g, 1 eq) was dissolved in dried *N,N*‐dimethylformamide (DMF) (55 ml) at 50°C and mixed with *N,N*‐diisopropylethylamine (DIPEA) (0.296 g, 20 eq), 1‐[bis(dimethylamino) methylene]‐1*H*‐benzotriazolium 3‐oxide hexa‐fluorophosphate (HBTU) (0.258 g, 6 eq), and VTX (0.474 g, 4.8 eq) in order. The mixed solution was stirred at 60°C for 6 h and then cooled to 40°C. The reaction solution was dropped into the methyl *tert*‐butyl ether (40 ml) at 30°C and let stand for 20 min. The suspension was slowly cooled to 0°C with stirring for 1.5 h. The precipitation was collected by filtration and washed with 20 ml of methyl *tert*‐butyl ether. The collected cake was dissolved in 20 ml of anhydrous ethanol in a flask at 40°C and mixed with 70 ml of methyl *tert*‐butyl ether. After incubation for 20 min, the suspension was cooled to 0°C with stirring for 1.5 h. The precipitation was again collected by filtration and washed with 20 ml of methyl *tert*‐butyl ether. These recrystallization processes were repeatedly carried out to obtain the final product, which was given the specification of a ≥97.0% PEG‐VTX compound with individual impurities ≤1.0%, as verified via HPLC analysis. The resultant cake was dried at 40°C under vacuum for more than 5 h, and the resultant yellowish powder product, PEG‐VTX (Figure 1), was stored in plastic bags at −20°C under a nitrogen atmosphere. The chemical structure of PEG‐VTX was analyzed via ^1^H‐NMR at 400 MHz (AVANCE III HD, Bruker). The purity of the PEG‐VTX was analyzed at a wavelength of 254 nm via HPLC (ACQUITY UPLC H‐Class, Waters Corporation) equipped with a 3.5 μm, 3.0 × 150 mm column of symmetry shield RP8, and eluted with a mixed solution of (A) 0.05% phosphate buffer and (B) acetonitrile with a 28‐min gradient condition as follows: 0 min; A/B = 60/40 (v/v), 6 min; A/B = 60/40 (v/v), 10 min; A/B = 40/60 (v/v), 20 min; A/B = 0/100 (v/v), 20 min; A/B = 60/40 (v/v), and 28 min; A/B = 60/40 (v/v). A flow rate of HPLC was 1.0 mL/min at 35°C.

### 2.3 Cancer cell lines

An MV4‐11 human hematopoiesis leukemia AML cell line (300295‐SF) was purchased from the Cell Lines Service. An OCI‐AML‐2 cell line was purchased from Nanjing Cobioer Gene Technology. A Panc‐1 human pancreas cancer cell line (RCB2095) and an A549 human lung cancer cell line (RCB0098) each was purchased from the RIKEN BioResource Center. The cells were obtained directly from each cell bank and were used for fewer than 6 months after receipt. The cells were cultured in RPMI‐1640 medium (FUJIFILM Wako Pure Chemical Corp.) supplemented with 10% heat‐inactivated fetal bovine serum, 100 units/ml penicillin, and 100 μg/ml streptomycin (ICN Biomedicals) in a 5% CO_2_/air incubator at 37°C.

### 2.4 In vitro cytotoxic assay

MV4‐11, Panc‐1, or A549 cells were seeded onto one of the wells of a 24‐well plate (1 × 10^5^ cells/well) containing an Opti‐MEM medium (Thermo Fisher Scientific) without FBS and incubated at 37°C overnight. The cells were exposed to VTX or PEG‐VTX at a VTX concentration of 1 pM ~ 100 μM (*n* = 3). After 48 h incubation, 50 μl of 3‐(4,5‐dimethylthiazol‐2‐yl)‐2,5‐diphenyltetrazolium bromide (MTT) solution (5 mg/ml) was added to the conditioned media, and the cells were further incubated for 2 h at 37°C. The cells were lysed with 300 μl of dimethyl sulfoxide (DMSO), and the absorbance at 540 nm in cell lysate was measured using a plate reader (Sunrise, Tecan).

### 2.5 In vitro measurement of protease activity in cancer cells

MV4‐11, Panc‐1, or A549 cells were harvested and collected into a tube (1 × 10^6^ cells/tube, *n* = 3). The cells were lysed with lysis buffer containing 1% Triton X‐100, 20 mM Tris–HCl, 137 mM NaCl, 10% Glycerol, and 1 mM EDTA. Then, the cell lysate was collected via centrifugation (10 000*×g*, 4°C, 10 min). The protein concentrations in the lysate were measured using a *DC* Protein Assay Kit (Bio‐Rad). The activity of proteases (trypsin, chymotrypsin, thermolysin, proteinase K, proteinase XIV, and elastase) in the resultant cell lysates was determined using an Amplite Universal Fluorimetric Protease Activity Assay Kit (AAT Bioquest).

### 2.6 In vitro measurement of the Bax expression level and cytochrome *c* in the cytoplasm and mitochondria of cancer cells

MV4‐11 cells were seeded into a T25 flask (2.5 × 10^6^ cells/flask) containing an Opti‐MEM medium and were incubated at 37°C overnight. The cells were incubated with VTX or PEG‐VTX at VTX concentrations of 0.01, 0.1, or 1 μM (*n* = 3). After 5 h incubation, both fractions of the cytoplasm and mitochondria were prepared and collected using a Cell Fractionation Kit (Abcam). The protein concentration of each lysate was measured using a *DC* Protein Assay Kit (Bio‐Rad). The expressions of Bax in the cytoplasmic and the mitochondrial fractions were determined using the automated capillary‐based electrophoresis Wes system (ProteinSimple), according to the manufacturer's instructions. The protein concentration was set to either 0.1 mg/ml for the cytoplasmic fraction or 0.15 mg/ml for the mitochondrial fraction. The expression of Bax was detected using an anti‐Bax primary antibody (ab32503; Abcam) at a dilution of 1:250. The expression of β‐Actin, as a loading control for cytoplasmic fraction, was detected using an anti‐β‐Actin primary antibody (ab16039; Abcam) at a dilution of 1:500. The expression of VDAC1/2, as a loading control for mitochondrial fraction, was detected using an anti‐VDAC1/2 primary antibody (10866‐1‐AP; Proteintech) at a dilution of 1:100. The antibody was further detected with a secondary HRP‐conjugated anti‐rabbit antibody (Anti‐Rabbit Detection Module, ProteinSimple). The assay was performed under the following conditions: Incubation with a primary antibody for 120 min and then a second incubation with a secondary antibody for 30 min.

The concentrations of cytochrome *c* in the cytoplasmic and mitochondrial fractions were measured using a Human Cytochrome *c* Quantikine ELISA Kit (R&D Systems) according to the manufacturer's instructions and corrected with these protein concentrations.

### 2.7 In vitro measurement of caspase activity in cancer cells

MV4‐11 cells were seeded onto one of the wells of a 96‐well plate (1 × 10^4^ cells/well) containing a reduced serum medium (Opti‐MEM) and were incubated at 37°C overnight. The cells were incubated with either VTX or PEG‐VTX at VTX concentrations of 0.001, 0.01, 0.1, 1, 10, or 100 nM (*n* = 6). After incubation for 24 h, the supernatants were collected, and the caspase activity in the supernatants was determined using a Caspase‐Glo 3/7 Assay System (Promega).

### 2.8 Animals and the in vivo Anti‐tumor effect of PEG‐VTX on tumor xenograft mice models

All the procedures related to animal handling, care, and treatment in this study were performed according to guidelines approved by the Institutional Animal Care and Use Committee (IACUC) of Pharmaron, Inc. following guidance from the Association for Assessment and Accreditation of Laboratory Animal Care (AAALAC). NOD SCID mice (female, 7–8 weeks old) and BALB/c *nu/nu* mice were purchased from Beijing AniKeeper Biotech Co., Ltd.. The experimental animals were allowed free access to water and mouse chow, and were housed under controlled environmental conditions (constant temperature and humidity, and a 12‐h dark–light cycle).

OCI‐AML‐2 and MV4‐11 tumor‐bearing mouse models were established by subcutaneous inoculations of OCI‐AML‐2 human acute myeloid leukemia cells (5 × 10^6^ cells/mouse) in the flanks of NOD SCID mice and inoculations of MV4‐11 cells (1 × 10^7^ cells/mouse) in the flanks of BALB/c *nu/nu* mice. All animal experiments were initiated when the tumors reached approximately 100 mm^3^ in size.

OCI‐AML‐2 tumor‐bearing mice were either injected with PEG‐VTX (100, 200, or 300 mg/kg/day, *i.v*.) once a week for 2 weeks or orally administered VTX (100 mg/kg/day, *p.o*.) every day for 2 weeks (*n* = 8). MV4‐11 tumor‐bearing mice were either injected with PEG‐VTX (300 mg/kg/day, *i.v*.) once a week for 3 weeks or orally administered VTX (50 mg/kg/day, *p.o*.) every day for 3 weeks (*n* = 8). The daily oral administration of VTX was performed using a feeding needle. Tumor volumes and body weights of the treated mice were recorded twice weekly. Tumor growth inhibition (TGI [%]) was calculated using the following formula (TV: tumor volume).^32^

TGI=1−TVTVcontrol×100

### 2.9 Statistical analysis

Statistical differences between the groups were evaluated via analysis of variance (ANOVA) with the Tukey *post‐hoc* test using Prism 8 software (GraphPad Software). All values are reported as the mean ± SD.

## 3 RESULTS

### 3.1 Synthesis and analysis of PEG‐VTX

PEG‐VTX was chemically synthesized by amide condensation of the carboxylic acid of each terminal end of 4‐armed PEG derivative (M.W. 40 000 Da) with the secondary amine of VTX (M.W. 868.44). The chemical structure of PEG‐VTX appears in Figure 1. The analytical identification of the chemical structure of PEG‐VTX was accomplished using ^1^H‐NMR. The signals in the solution of DMSO‐d_6_ were 0.92 (s, 24H), 1.23 (m, 8H), 1.39 (m, 8H), 1.60 (m, 8H), 1.86 (m, 4H), 1.96 (brs, 8H), 2.20 (m, 24H), 2.79 (brs, 8H), 3.50 (m, CH_2_ of PEG), 3.87 (m, 8H), 5.16 (s, 8H), 6.40 (m, 4H), 6.68 (m, 4H), 6.77 (m, 4H), 7.05 (m, 12H), 7.34 (m, 8H), 7.44 (m, 4H), 7.51 (m, 4H), 7.75 (m, 4H), 7.97 (m, 4H), 8.06 (m, 4H), 8.43 (m, 4H), 8.51 (m, 4H), and 11.7 (brs, 1H). The purity of PEG‐VTX was analyzed via HPLC, which showed one single strong peak at 8.026 with no other signals. These results indicate that the synthesized PEG‐VTX was composed of 4 mol VTX with 1 mol PEG derivative (M.W. 43 474 Da) and had a high level of purity.

### 3.2 In vitro cytotoxicity on cancer cells of either free VTX or PEG‐VTX

**FIGURE 2:**
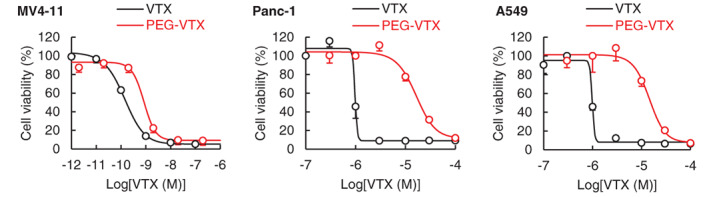
Cytotoxic effects of either VTX or PEG‐VTX on three different human cancer cell lines in vitro. MV4‐11, Panc‐1, or A549 cells were seeded onto one of the wells of a 24‐well plate (1 × 10^5^ cells/well) and were exposed to either free VTX or PEG‐VTX (1 pM ~ 100 μM as a VTX concentration). After 48 h of incubation, cell viability was determined via MTT assay. The plot data are reported as the means ± SD (*n* = 3), and the lines are a fitted curve calculated using the plot data

The levels of cytotoxicity of both free VTX and PEG‐VTX were individually evaluated using MV4‐11 human AML cells, Panc‐1 human pancreas cancer cells, and A549 human lung cancer cells (Figure 2). To exclude the effect of protein binding, an Opti‐MEM medium without the addition of FBS was used because free VTX easily binds to serum proteins (>99.9%).^16^ The concentration that was required in order to produce 50% inhibitory effects (IC_50_) appears in Table 1. In the MV4‐11 cells, PEG‐VTX inhibited cellular growth, as shown in the left panel of Figure 1, on an almost equal level with free VTX. Interestingly, in the Panc‐1 and A549 cells, the IC_50_ values of PEG‐VTX were much higher (19 times and 16 times, respectively) than those of free VTX (middle and right panels of Figure 2).

**TABLE 1:** IC_50_ of free VTX and PEG‐VTX on three different cancer cell lines

Treatment	MV4‐11	Panc‐1	A549
VTX	0.00016 μM	0.99 μM	0.99 μM
PEG‐VTX	0.00085 μM	18.9 μM	15.5 μM

**FIGURE 3:**
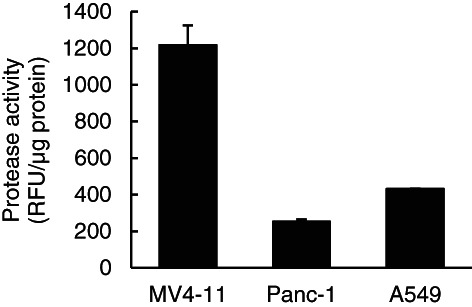
Protease activities in three different human cancer cell lines in vitro. MV4‐11, Panc‐1, or A549 cells were collected into a tube (1 × 10^6^ cells/tube) and were lysed with lysis buffer. The activity of proteases (trypsin, chymotrypsin, thermolysin, proteinase K, proteinase XIV, and elastase) in the prepared lysates then were determined using an Amplite Universal Fluorimetric Protease Activity Assay Kit, and were corrected using these protein concentrations. The data represent the means ± SD (*n* = 3)

To gain insight into the mechanism of PEG‐VTX and the superior cytotoxicity to MV4‐11 cells, as shown in Figure 2, the activities of the global proteases in each cell line were determined for their level of involvement in VTX release from the conjugates (Figure 3). The level of protease activity was 3–4 fold larger in MV4‐11 cells than in either Panc‐1 cells or A549 cells.

### 3.3 Changes in Bax expression in cytoplasm and mitochondria by treatment with either free VTX or PEG‐VTX in vitro

**FIGURE 4:**
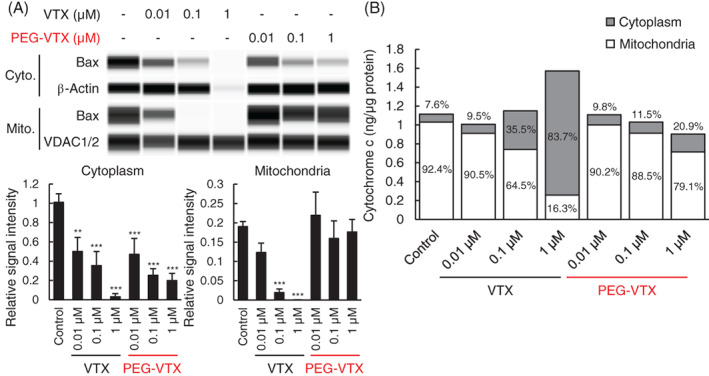
Bax expression levels and cytochrome *c* concentrations in the cytoplasm or mitochondria following treatment with either free VTX or PEG‐VTX in vitro. MV4‐11 cells were seeded into a T25 flask (2.5 × 10^6^ cells/flask) and were exposed to either free VTX or PEG‐VTX (0.01, 0.1, or 1 μM as an API VTX concentration). After 5 h of incubation, the cytoplasm and mitochondria fractions were collected. (A) The expression levels of Bax and β‐Actin in cytoplasm fraction and those of Bax and VDAC1/2 in mitochondria fraction were determined using a Wes system. The lower graphs show the relative signal intensity of Bax corrected by that of β‐Actin for cytoplasm fraction or that corrected by VDAX1/2 for mitochondria fraction. The data represent the mean ± SD (*n* = 3, **p < .01, ***p < .001 vs. control). (B) The concentrations of cytochrome *c* in these fractions was measured via ELISA, and corrected using these protein concentrations. The data represent a typical result from three independent experiments

Bax is a member of the Bcl‐2 family that accelerates apoptosis and is predominantly present in the cytoplasm of cells.^2^ A recent study reported that Bax redistributes from the cytosol to membranes via Bcl‐2‐mediated apoptosis signaling, and this redistribution of Bax is inhibited by treatment with cycloheximide, which is an apoptosis inhibitor.^33^ Thus, as an indication of Bcl‐2‐mediated apoptosis, the expressions of Bax in the cytoplasm and mitochondria were studied following treatment of MV4‐11 cells with either free VTX or PEG‐VTX (Figure 4A upper panel). The unedited raw data of expressions of Bax and β‐Actin in cytoplasm is shown in Figure S1, and that of Bax and VDAC1/2 in mitochondria is shown in Figure S2. The signal intensity of each bands was quantified and represented as a relative signal intensity corrected by the expression of a loading control, β‐Actin for cytoplasm or VDAC1/2 for mitochondria (Figure 4A lower panels). The treatment with free VTX decreased the expression of Bax in the cytoplasm in a dose‐dependent manner, as well as that in the mitochondria. The treatment with PEG‐VTX consistently decreased the expression of Bax in the cytoplasm in a dose‐dependent manner, but not in the mitochondrial expression of Bax. This finding indicates that PEG‐VTX induces a redistribution of Bax from the cytoplasm to a membrane and causes Bcl‐2‐mediated apoptosis, similar to the results achieved using free VTX.

### 3.4 Effusion of cytochrome *c* from mitochondria to the cytoplasm by treatment with either free VTX or PEG‐VTX in vitro

Cytochrome *c* is an indispensable factor for inducing Bcl‐2‐mediated apoptosis.^34^ It is well known that cytochrome *c* is localized in mitochondria of normal cells and released to the cytoplasm while inducing a signaling cascade of apoptosis.^35^ In order to confirm the effusion of cytochrome *c* from mitochondria as another indication of Bcl‐2‐mediated apoptosis, the amounts of cytochrome *c* in the fractions of both mitochondria and the cytoplasm were measured following treatment with either free VTX or PEG‐VTX (Figure 4B). Treatment with free VTX induced decreases in the amount of cytochrome *c* in mitochondria and increases in the cytoplasm in a dose‐dependent manner. Treatment with PEG‐VTX also induced decreases in the amount of cytochrome *c* in mitochondria and increases in the cytoplasm in a dose‐dependent manner, although the impact on cytochrome *c* changes in both the cytoplasm and mitochondria were much stronger with free PTX treatment than with PEG‐VTX treatment.

### 3.5 Activation of caspase by treatment with either free VTX or PEG‐VTX in vitro

**FIGURE 5:**
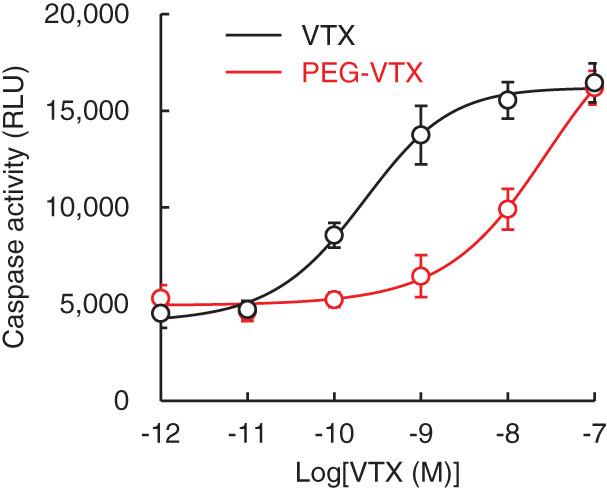
Caspase activity after treatment with either free VTX or PEG‐VTX in vitro. MV4‐11 cells were seeded onto one of the wells of a 96‐well plate (1 × 10^4^ cells/well) and were incubated with either free VTX or PEG‐VTX (0.001, 0.01, 0.1, 1, 10, or 100 nM as a VTX concentration). After 24 h of incubation, the caspase activity in the media was determined. The plot data are reported as the means (*n* = 6 in each concentration point), and the lines were a fitted curve calculated using the plot data

The activity of caspase, a critical apoptotic factor, was evaluated after treatment with either free VTX or PEG‐VTX (Figure 5). Treatment with free VTX increased caspase activity with an increase in the VTX dosage. Treatment with PEG‐VTX also increased caspase activity in a dose‐dependent manner. The activities of free VTX with respect to increases in caspase activity; however, were two orders of magnitude higher than that of PEG‐VTX. On the other hand, at a higher concentration (0.1 μM), PEG‐VTX promoted high caspase activity in MV4‐11 AML cells at a rate that was equivalent to that of free VTX. These results suggest that PEG‐VTX can induce apoptosis via promoting apoptosis signals in AML cells.

### 3.6 In vivo anti‐tumor effects of either free VTX or PEG‐VTX on two different AML tumor‐bearing mouse models

**FIGURE 6:**
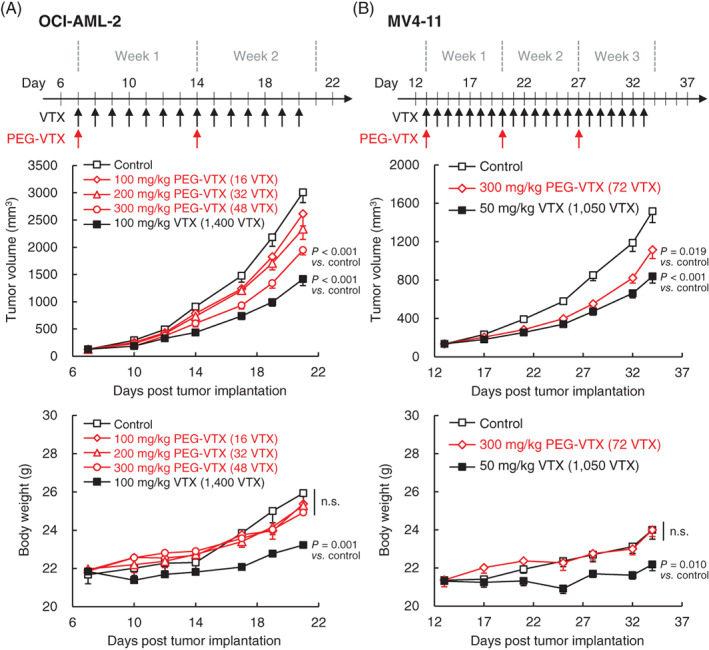
In vivo anti‐tumor effects of either free VTX or PEG‐VTX on either an OCI‐AML‐2 xenograft mouse model or a MV4‐11 xenograft mouse model. (A) In the PEG‐VTX treatment group, OCI‐AML‐2 tumor‐bearing mice were intravenously injected with PEG‐VTX (100, 200, or 300 mg/kg/day) once a week for 2 weeks. In the free VTX treatment group, doses of free VTX (100 mg/kg/day) were orally administered every day for 2 weeks. (B) In the PEG‐VTX treatment group, MV4‐11 tumor‐bearing mice were intravenously injected with PEG‐VTX (300 mg/kg/day) once a week for 3 weeks. In the free VTX treatment group, free VTX (50 mg/kg/day) were orally administered every day for 3 weeks. Tumor growth and body weights were recorded twice weekly. The data represent the means ± SD (*n* = 8, n.s., not significant)

The in vivo anti‐tumor effects of PEG‐VTX were studied in both OCI‐AML‐2 and MV4‐11 xenograft mouse models. In the OCI‐AML‐2 xenograft model, the clinical dosage was mimicked by orally administering free VTX daily for 2 weeks at a dose of 100 mg/kg/day: the total VTX dosage was 1400 mg/kg (Figure 6A upper panel). PEG‐VTX was intravenously administered at 100, 200 or 300 mg/kg once a week for 2 weeks: the total VTX dosages were 16, 32, 48 mg/kg since theoretically 100 mg of PEG‐VTX contains 8 mg of VTX as an active pharmaceutical ingredient (API). Intravenous PEG‐VTX showed a somewhat weaker growth‐inhibitory effect even at the highest dose (300 mg/kg), compared with 100 mg/kg of oral free VTX (Figure 6A middle panel and Table 2). The doses of converted API and VTX, however, were much smaller in PEG‐VTX (48 mg/kg) than in free VTX (1400 mg/kg). In addition, and most important, the treatments of intravenous PEG‐VTX even at the highest dose (300 mg/kg) caused no body weight loss of the treated mice, while the treatments of oral free VTX (100 mg/kg) caused >16% body weight loss (Figure 6A lower panel). Similar experiments with different dosage regimens were conducted with the MV4‐11 xenograft model (Figure 6B). Free VTX (50 mg/kg) was orally administered daily for 3 weeks (i.e., total VTX dose 1050 mg/kg), while PEG‐VTX (300 mg/kg) was intravenously administered once a week for 3 weeks (i.e., total API VTX dose 72 mg/kg) (Figure 6B upper panel). The intravenous PEG‐VTX clearly inhibited tumor growth comparable to that of oral free VTX (Figure 6B middle panel and Table 3). In addition, the intravenous PEG‐VTX caused no body weight loss, while the oral free VTX caused >10% body weight loss (Figure 6B lower panel). These results indicate that PEG‐VTX has the potency to treat AML tumors without inducing systemic side effects.

**TABLE 2:** TGI of free VTX and PEG‐VTX on OCI‐AML‐2 xenograft mouse model

Treatment	TGI (%)
100 mg/kg PEG‐VTX (16 VTX)	13.1
200 mg/kg PEG‐VTX (32 VTX)	22.4
300 mg/kg PEG‐VTX (48 VTX)	35.2
100 mg/kg VTX (1400 VTX)	52.9

**TABLE 3:** TGI of free VTX and PEG‐VTX on MV4‐11 xenograft mouse model

Treatment	TGI (%)
300 mg/kg PEG‐VTX (72 VTX)	26.5
50 mg/kg VTX (1050 VTX)	44.7

## 4 DISCUSSION

In the current study, we successfully synthesized a PEG‐drug conjugate of the marketed anticancer drug VTX (Figure 1) and confirmed that PEG‐VTX promotes the cytotoxicity of MV4‐11 human AML cells. This was not the case, however, for other cancer cells such as Panc‐1 human pancreas cancer cells and A549 human lung cancer cells in vitro (Figure 2 and Table 1). The original application of VTX was as a BH3 mimetic highly selective for Bcl‐2.^15^ The database of the Cancer Cell Line Encyclopedia (CCLE) produced by the Broad Institute of MIT and Harvard (https://portals.broadinstitute.org/ccle) indicates the relative expression level of *BCL2* mRNA is much higher in AML cells (3.69) than in either pancreas cancer cells (−2.06) or in nonsmall cell lung cancer (NSCLC) cells (−1.54). In addition, VTX is known to exhibit a higher level of sensitivity to the T‐cell acute lymphoblastic leukemia cell lines in correspondence with a higher expression level of Bcl‐2 proteins.^36^ Indeed, our series of in vitro studies showed that PEG‐VTX induces cytotoxicity via the promotion of Bcl‐2‐mediated apoptosis (Figures 4, 5, and 6) in the same manner as the original VTX,^15^, ^37^ which means that PEGylation to VTX did not affect its original cytotoxic properties.

In our PEG‐VTX conjugates, VTX and 4‐armed PEG derivatives were covalently attached through an amide bond linker (Figure 1). PEGylation of anticancer drugs is well recognized as a promising strategy to improve water solubility, prolong blood circulation time, minimize undesired side effects by avoiding nonspecific distribution in the body, and improve tumor accumulation via the EPR effect.^22^ At the same time, PEGylation tends to hinder the penetration of attached drugs into cancer cells due to the increased hydrodynamic sizes of conjugates that correspond to their increased molecular weight. To gain insight into the mechanisms that promote the cytotoxicity of PEG‐VTX, in the present study, we determined the activities of global proteases (trypsin, chymotrypsin, thermolysin, proteinase K, proteinase XIV, and elastase) in the cells, because these are directly involved in the release of VTX from the conjugates. As shown in Figure 3, MV4‐11 human AML cells showed the highest level of sensitivity to PEG‐VTX (Figure 2 and Table 1) as well as a higher level of protease activity compared with that of either Panc‐1 human pancreas cancer cells or A549 human lung cancer cells. Those results support our original thesis that proteases inside/outside cancer cells enzymatically cleave the amide bond linkers between VTX and 4‐armed PEG derivatives, and the derived active ingredient VTX is finally produced to induce apoptosis in AML cells. Nevertheless, changes in the expression of Bax proteins (Figure 4A), the effusion of cytochrome *c* (Figure 4B), and the activation of caspase (Figure 5) all are related to the signaling cascade of apoptosis and all were present in much higher levels in cells treated with free VTX than in those treated with PEG‐VTX in vitro. This is probably due to a shorter incubation time for PEG‐VTX to reach a sufficient concentration of the active ingredient VTX in order to induce apoptosis relative to free VTX, because the enzymatic cleavage‐mediated release of VTX from PEG‐VTX was expected to be quite slow.

The 4‐armed PEG derivatives that were used to synthesize PEG‐VTX have been used for several clinical studies in conjugation with an anti‐cancer agent.^38^ PEGylated SN38 conjugates (EZN‐2208) are composed of 4‐armed PEG derivatives with a glycine spacer,^23^ and these have shown therapeutic effects in patients with neuroblastoma^24^ and acute promyelocytic leukemia.^25^ PEGylated irinotecan conjugates (NKTR‐102) are composed of 4‐armed PEG derivatives with a cleavable ester‐based linker,^39^ and have achieved prolonged systemic exposure of SN38, an active metabolite of irinotecan, and shown therapeutic effects on metastatic breast cancer,^26^ epithelial ovarian cancer,^27^ high‐grade glioma,^28^ and metastatic NSCLC^29^ in patients in a clinical setting. It is unfortunate, however, that these PEG‐drug conjugates could not meet their primary endpoints. These kinds of PEG‐drug conjugates are designed to quickly release the active drugs at a target cite by chemically attaching the drugs to PEG derivatives through cleavable linkages such as either ester bonds or amide‐oxy bonds, which are cleaved by a common reaction with metabolic enzymes such as esterase or carboxylase.^40^ Such metabolic enzymes are present in the body everywhere, which includes blood, so the PEG‐drug conjugates immediately release the active drugs in blood after intravenous injection/infusion and consequently lose the designed therapeutic effect. Instead, the drugs actually induce undesired systemic side effects due to nonspecific distribution in the body. This could be a major reason that PEG‐drug conjugates were withdrawn from clinical trials. In contrast, our PEG‐VTX was synthesized through an amide bond to covalently bind VTX to the end of 4‐armed PEG derivatives that are well‐known to be stable in the human body for extended periods of time. The PEG‐VTX delivers sufficient amounts of the active ingredient VTX to solid tumors via the EPR effect owing to a very slow release in the blood. In solid tumors, accumulated PEG‐VTX releases VTX in the tumor microenvironment through enzymatic cleavage by proteases specifically overexpressed in the tumor. *N*‐acylethanolamine‐hydrolyzing acid amidase (NAAA), an amidase acting at acidic pH,^41^ is reported to be highly expressed in several types of cancer cells.^42^ The results of the present study have shown sufficient tumor‐growth inhibition by PEG‐VTX in AML tumor‐xenograft mouse models without severe systemic side effects (Figure 6).

Most importantly, intravenous PEG‐VTX caused no body weight loss and sufficient tumor growth suppression, while oral free VTX caused significant body weight loss with tumor growth suppression that was more potent than that of PEG‐VTX (Figure 6). In our series of in vivo experiments, the total dosages of API and VTX were 15 or 30 times smaller in a PEG‐VTX treatment group than in the free VTX treatment group. Although an oral administration route is preferred over various other administration routes of drug delivery, low/poor bioavailability of administered drugs is the largest problem for oral administration. In clinical settings, free VTX is administered to patients with a cumbersome daily or weekly ramp‐up schedule,^16^ because of low bioavailability and severe side effects. It is plausible that such a complicated regimen would decrease patient compliance. Although intravenous injection/infusion of drugs is highly invasive, the bioavailability must be defined as 100%. Owing to increased water solubility and to the hydrodynamic size of VTX via PEGylation, intravenous PEG‐VTX is reliably assumed to have properties that extend blood circulation time, which results in a higher accumulation in solid tumors via the EPR effect with less distribution in normal tissue. Regarding the PEGylated irinotecan (NKTR‐102), plasma concentrations of NKTR‐102 are highly sustained in both rat and dog after the intravenous injection compared to irinotecan free form,^39^ which speculates that our PEG‐VTX could sustain in blood circulation higher than VTX free form. This drastic improvement in the pharmacokinetics of VTX by PEGylation in this study should allow decreases in the injection dosages of API, VTX, as well as a decrease in the dosing frequency, although further investigations into the pharmacokinetics of PEG‐VTX in animal models will be necessary.

## 5 CONCLUSIONS

In the present study, a novel PEG‐drug conjugate, PEG‐VTX, was synthesized and showed highly cytotoxic effects on AML cells both in vitro and in vivo. Most importantly, intravenous PEG‐VTX induced sufficient tumor growth suppression without severe adverse side effects with a dosage regimen that was 20‐fold less than the total doses of oral free VTX, which induces a somewhat higher level of tumor growth inhibition at the cost of a greater amount of body weight loss. PEG‐VTX could be an alternative therapeutic option that is safe and effective for the treatment of patients with AML.

## CONFLICT OF INTEREST

Kiyoshi Eshima is President of Delta‐Fly Pharma, Inc. The other authors have no potential conflicts of interest in this study.

## AUTHOR CONTRIBUTIONS

**Hidenori Ando:** Conceptualization; formal analysis; funding acquisition; investigation; methodology; project administration; validation; visualization; writing‐original draft; writing‐review & editing. **Yuta Murakami:** Investigation; resources; writing‐original draft; writing‐review & editing. **Kiyoshi Eshima:** Conceptualization; project administration; resources; supervision; visualization; writing‐original draft; writing‐review & editing. **Tatsuhiro Ishida:** Conceptualization; formal analysis; funding acquisition; investigation; methodology; project administration; supervision; validation; visualization; writing‐original draft; writing‐review & editing.

## ETHICS STATEMENT

All the procedures related to animal handling, care, and treatment in this study were performed according to guidelines approved by the Institutional Animal Care and Use Committee (IACUC) of Pharmaron, Inc. (Beijing, China) following guidance from the Association for Assessment and Accreditation of Laboratory Animal Care (AAALAC).

## Supporting information


**Figure S1.** Unedited Wes data on Bax expression in cytoplasm
**Figure S2.** Unedited Wes data on Bax expression in mitochondria

## Data Availability

The data for this report are available from the corresponding author on reasonable request.
