# Supplementary material for: A novel polyethylene glycol (PEG)‐drug conjugate of Venetoclax, a Bcl‐2 inhibitor, for treatment of acute myeloid leukemia (AML)
Source: Cancer Rep (Hoboken). 2021 Jun 26;5(3):e1485. doi: 10.1002/cnr2.1485 (PMC8955075; doi:10.1002/cnr2.1485)
Supplement: Supplementary file 1 — Figure S1. Unedited Wes data on Bax expression in cytoplasm Figure S2. Unedited Wes data on Bax expression in mitochondria [file CNR2-5-e1485-s001.pptx]

## Slide 1
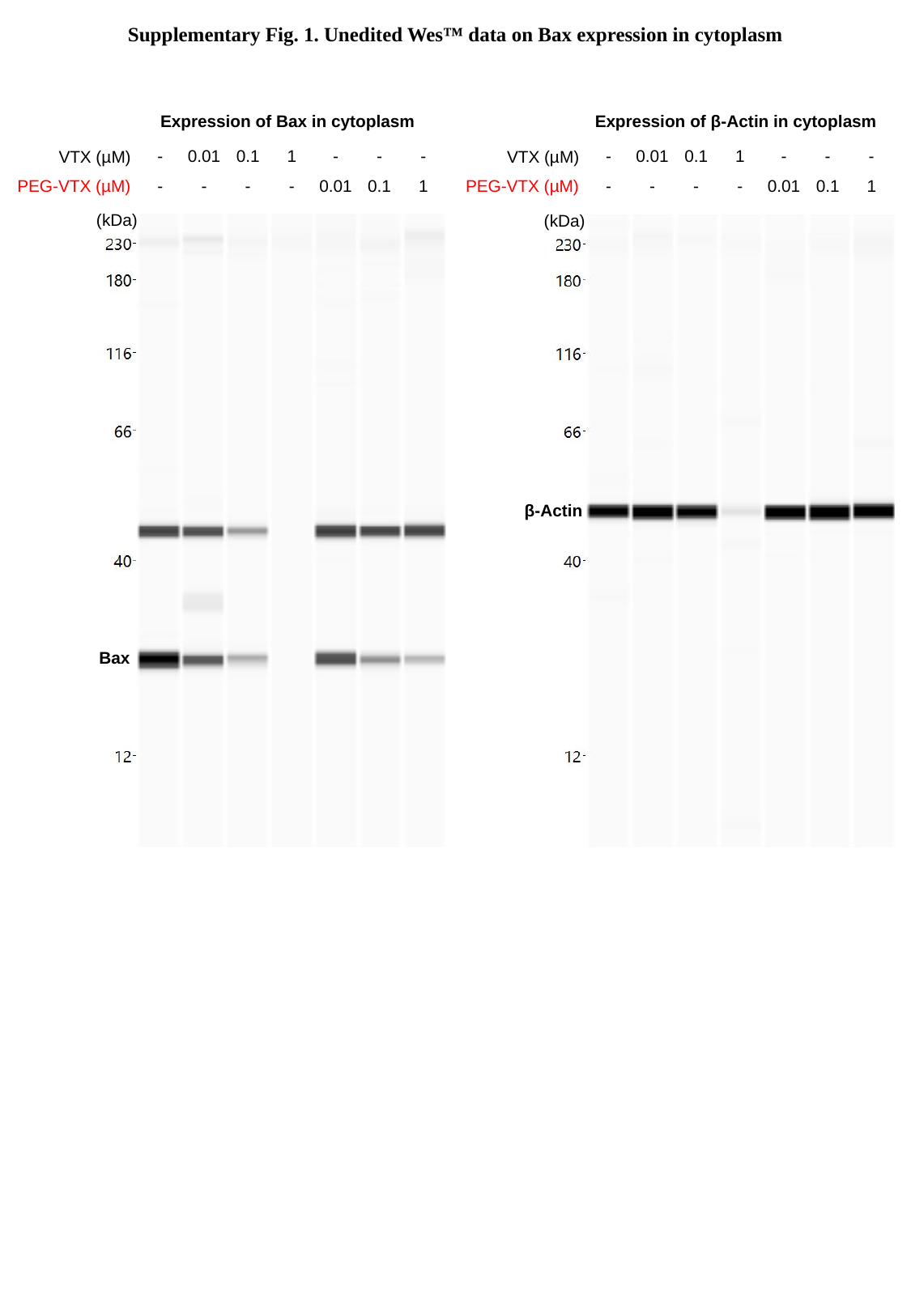

Supplementary Fig. 1. Unedited Wes™ data on Bax expression in cytoplasm
Expression of Bax in cytoplasm
Expression of β-Actin in cytoplasm
-
0.01
0.1
1
-
-
-
-
0.01
0.1
1
-
-
-
VTX (µM)
VTX (µM)
-
-
-
-
0.01
0.1
1
-
-
-
-
0.01
0.1
1
PEG-VTX (µM)
PEG-VTX (µM)
(kDa)
(kDa)
β-Actin
Bax

## Slide 2
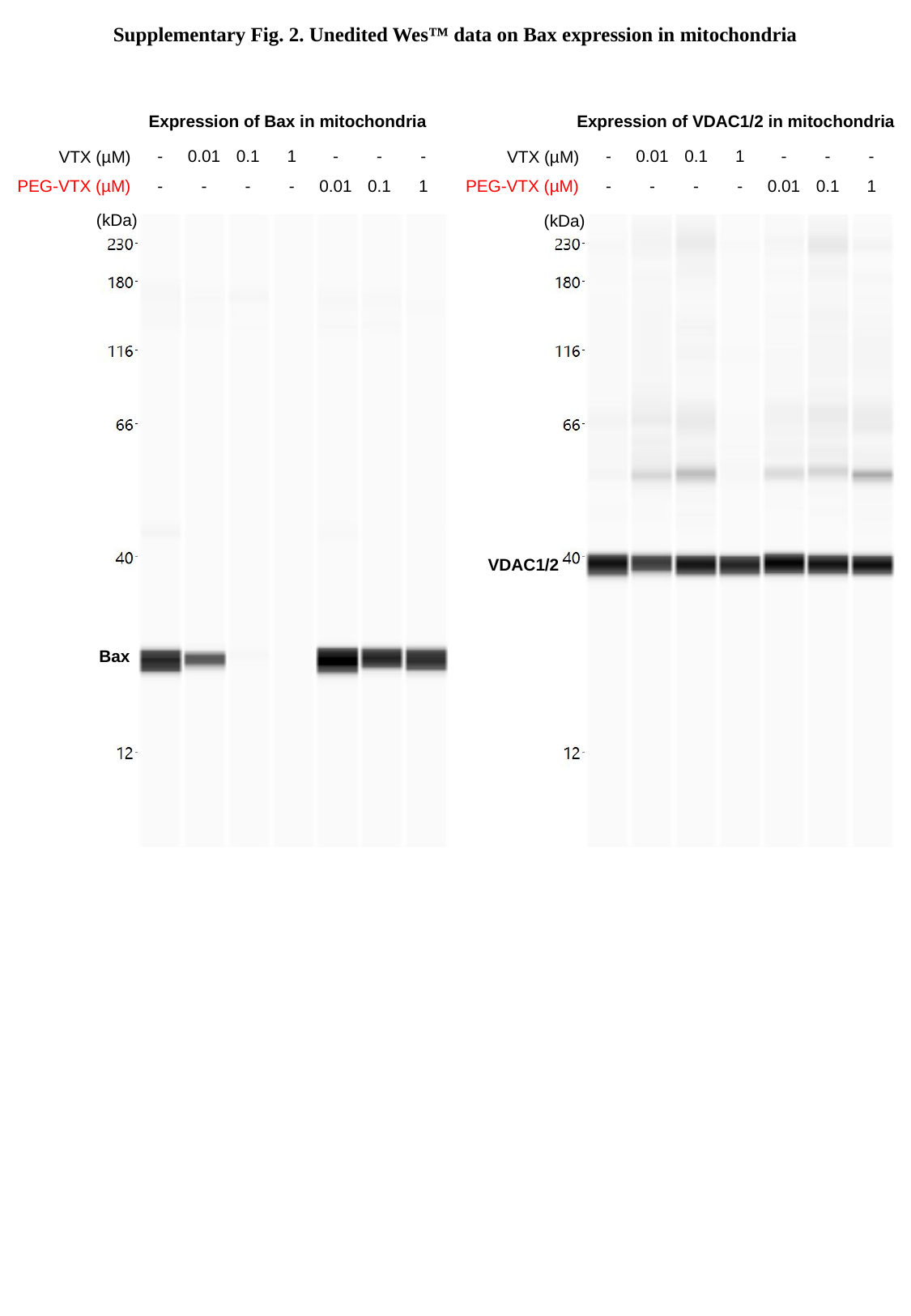

Supplementary Fig. 2. Unedited Wes™ data on Bax expression in mitochondria
Expression of Bax in mitochondria
Expression of VDAC1/2 in mitochondria
-
0.01
0.1
1
-
-
-
-
0.01
0.1
1
-
-
-
VTX (µM)
VTX (µM)
-
-
-
-
0.01
0.1
1
-
-
-
-
0.01
0.1
1
PEG-VTX (µM)
PEG-VTX (µM)
(kDa)
(kDa)
VDAC1/2
Bax
